# Bridging gaps between scientific research and regulatory decision-making in Europe: roles of academia, risk assessors, and policymakers

**DOI:** 10.3389/ftox.2026.1829326

**Published:** 2026-05-04

**Authors:** Ana Fernandez-Agudo, Hannah Lester, Jose V. Tarazona, Fernando Rivero-Pino

**Affiliations:** 1 Spanish National Environmental Health Center, Instituto de Salud Carlos III, Madrid, Spain; 2 Atova Regulatory Consulting, SLU, Barcelona, Spain

**Keywords:** experimental design, fit-for-purpose, policymaking, research integrity, scientific communication

## Abstract

A gap between scientific research and regulatory frameworks continues to challenge the effective translation of science into policy and regulation. This commentary explores the disconnection between scientific research, regulatory agencies, and policymakers, highlighting the distinct yet interdependent roles they play. Researchers generate data, which is used by regulatory agencies for risk assessment, and these outcomes serve as the basis for policy development by policymakers. However, misalignments between these key stakeholders hinder effective integration, with consequences for advancing technological development. Additionally, it proposes a set of practical and strategic solutions that span academia, regulatory science, and policymaking to aid the effective integration of science into policy and regulation. Regulatory frameworks that encourage data harmonization, new options for validation, and accessibility are needed. Funding mechanisms that incentivize responsible and relevant research practices are also emphasized. Finally, the importance of open communication and targeted training for researchers and regulators to bridge knowledge gaps and build trust is also discussed. By addressing these issues, this manuscript aims to strengthen the synergy between scientific developments and regulation, advancing evidence-based policymaking. Enhancing robust, reliable and relevant science would improve regulatory decision-making processes, while also guiding researchers on responsible methodologies and setting standards for future research recommendations. The paper advocates for a collaborative approach to enhance the relevance and reliability of published research in guiding evidence-based regulation and policy.

## 1 Introduction

While most legislative acts emphasize the importance of incorporating scientific knowledge into decision-making processes, its implementation in practice often falls short. Scientific Committees and regulatory agencies were established to bridge this gap by providing science-based assessments to inform policy. However, despite their efforts, a persistent disconnect remains between the generation of scientific data, risk assessments, and the development of policies (Kano and Hayashi, 2021). This gap between scientific research and regulatory frameworks poses significant challenges for translating research findings into effective policies.

Academia is the main mechanism for advancing scientific knowledge through fundamental and applied research, generating data and results that often serve as the foundation for innovation and, ideally, policy development. Risk assessors, typically operating within regulatory agencies, evaluate scientific evidence to determine the safety, quality, and efficacy of products, processes, or practices. They employ standardized methodologies and ensure compliance with legal frameworks, translating scientific findings into actionable guidelines and assessments. Many agencies and regulatory bodies complement their scientific staff with scientific committees and panels of external experts including active researchers. Policymakers, on the other hand, are tasked with designing and implementing laws, regulations, and policies that address societal challenges. Their decisions rely heavily on the evidence provided by both academia and risk assessors to ensure that policies are scientifically robust, and also economically viable and socially acceptable (McKenna, 2021).

The relationship among these stakeholders is interdependent: academia provides the evidence, risk assessors bridge the gap by contextualizing and standardizing this data, and policymakers utilize these outputs to develop and enforce regulations, however, scientific research frequently struggles to effectively address regulatory needs, which can hinder its potential to support evidence-based decision-making. While science is expected to extend beyond regulatory requirements, ensuring that scientific knowledge is readily applicable for informed policymaking is essential to advancing public health and fostering innovation.

Misaligned priorities, methodological flaws, and communication barriers hinder the practical application of scientific advancements; addressing this disconnect requires a critical examination (Starokozhko et al., 2021).

Firstly, research, especially in academia, is pressured by the publish or perish game - in which citations and number of publications is the main goal of research. This may misrepresent public understanding and complicate regulatory interpretation, as suggested in previous analyses of science communication and research integrity (Herndon, 2016). This hinders practical policy implementation, and affects public perception, regulatory efficacy, and the overall credibility of scientific inquiry. Such distortions could lead to misinformed regulatory actions, either imposing unnecessary restrictions or failing to address potential risks adequately. In fact, it has been noted the increase in the number of papers retracted in recent years, reflecting growing concerns about research integrity and the prevalence of misconduct or errors in scientific work. In this context, there is a clear need for a more accurate scientific execution and reporting of data, as well as collection of data and homogenization of the methods (Dwivedi et al., 2024) to increase the extent to which their work aligns with regulatory needs.

Secondly, risk assessors are responsible for translating scientific research into regulatory frameworks, ensuring that regulatory decisions are based on comprehensive, evidence-based assessments that maintain the credibility of the regulatory process. However, their ability to do so is shaped by legal mandates, resource limitations, and the need to ensure consistency within established regulatory systems. Previous experience is transferred into guidance to facilitate new assessments. However, fixing methodologies and approaches can sometimes contribute to widening the gap between scientific developments and regulation. Researchers working as risk assessors in expert panels often rely on rigid, standardized methodologies that may not always accommodate emerging scientific approaches. Too strict adherence to predefined risk models and reliance on conservative safety factors (used to account for variability and data gaps) can, in some cases, limit the integration of emerging scientific approaches when evidence is scarce or not yet standardized. It is important to note that these uncertainty factors are essential components of risk assessment frameworks, designed to ensure protection in the presence of limited data. However, challenges arise when there are insufficient data to refine these factors, raising the question of how to balance precaution with the integration of innovative methodologies. To address this challenge, several approaches can be considered: (i) the use of data-derived or chemical-specific adjustment factors where sufficient evidence exists; (ii) the integration of probabilistic risk assessment methods to better characterise uncertainty; and (iii) the use of weight-of-evidence frameworks to incorporate diverse data streams, including NAMs, even when full validation is pending. These approaches do not reduce safety margins *per se* but allow for their refinement and contextualisation. Additionally, some regulatory frameworks disconnect the assessment of potential risks from the expected benefits, creating regulatory hurdles that slow down scientific advancements. While their work is intended to ensure safety, the cautious nature of their evaluations can inadvertently contribute to regulatory inertia. To overcome that, a Safe and Sustainable by Design (SSbS) approach offers a proactive solution by integrating safety, sustainability, and functionality considerations early in the innovation process, fostering regulatory compliance while accelerating the responsible development of novel technologies. This approach enables risk assessors to work proactively with researchers, ensuring that new materials, chemicals, and processes are designed to meet safety and environmental standards from the outset. As a result, regulatory evaluations can become more adaptive and dynamic, reducing delays while maintaining high safety standards (Centre et al., 2022).

On the other hand, risk assessors serving as experts in scientific committees (e.g., Risk Assessment Committee (RAC) at the European Chemicals Agency (ECHA) or at the European Food Safety Authority (EFSA) (2025), may be required by the regulatory framework to prioritize regulatory consistency over scientific progress, reducing the capacity for adopting new methodologies that challenge pre-established norms. Instead of fostering innovation, the regulatory approach may reinforce existing barriers, making it more difficult for emerging scientific developments and better alternatives to gain regulatory acceptance. Additionally, some regulatory frameworks separate risk assessment from socio-economic considerations, as observed in systems where committees such as the Risk Assessment Committee (RAC) and the Socio-Economic Analysis Committee (SEAC) operate with distinct mandates. While this separation ensures scientific independence, it may also lead to situations where risk characterisation and benefit considerations are not fully integrated in early stages of decision-making. To ensure that regulatory frameworks remain robust without stifling innovation, risk assessors must actively engage in promoting adaptive methodologies, understood as approaches that allow the iterative integration of new data, the use of tiered testing strategies, and the incorporation of non-traditional evidence streams (e.g., NAMs, *in silico* models, and real-world data) within existing regulatory frameworks (Pascarella et al., 2021; Vareman and Persson, 2010).

Thirdly, policymakers are expected to promote homogenized, transparent data-sharing platforms; however, their capacity to implement such measures is often constrained by political priorities, competing interests, and institutional inertia. Balancing the need for standardized protocols with the integration of innovative approaches and their data into regulatory systems (Anastassiadou et al., 2025). This process can be facilitated by pre-validation steps, such as setting standard operating procedures, and clear validation pathways. Without these frameworks, scientifically valid emerging technologies often struggle to gain acceptance, delaying their integration into regulatory practice. This is particularly evident in the work of regulatory committees, which could serve as important platforms to encourage the use and validation of new methods. Given the scientific and legal requirement to demonstrate an acceptable level of risk, flexibility should not be interpreted as safety standards or regulatory thresholds with lower requirements. Rather, flexibility refers to the processes leading to these conclusions, including the types of evidence considered, the methodologies applied, and the timing of their integration. This may include accepting alternative data sources, applying tiered approaches, or provisionally considering emerging methodologies alongside traditional evidence, while maintaining the same level of protection. Otherwise, if methods are only validated after lengthy processes, they risk becoming outdated by the time they are accepted, while newer, more relevant methods are overlooked. Moreover, funding mechanisms should prioritize transparency and methodological rigor by incentivizing open data practices and harmonized research approaches. This shift would encourage researchers to align their work with regulatory standards (Prata et al., 2021), even if the laboratory is not formally under the regulatory scheme. These approaches would ultimately improve the reliability of scientific findings (Freudenthal et al., 2024; Scholz et al., 2022). Finally, open communication and targeted training for both researchers and regulators are vital. Regular dialogue, advisory panels, and comprehensive education initiatives can bridge knowledge gaps, improve regulatory assessments, and foster mutual understanding. By implementing these strategies, policymakers can strengthen evidence-based decision-making, enhance public trust, and ensure that scientific development effectively informs regulation. The Joint Research Centre (JRC) of the European Commission launched in September 2020 the science-for-policy ecosystems, strengthening and connecting science for policy ecosystems across the European Union and within its Member States (European Commission, 2020). From this new partnership, an evaluation framework to assess the capacity of science for policy ecosystems in EU Member States started to develop at the end of 2021. This means that a transition towards a more efficient and connected risk assessment is already taking place.

While previous studies have examined the science-policy interface and the integration of research into regulatory decision-making (Hofmann et al., 2025; Reichmann and Wieser, 2022; Suazo-Galdames et al., 2025; Zare Jeddi et al., 2023), much of the existing literature focuses either on high-level frameworks or on specific sectors. Less attention has been given to the practical misalignments that occur across academia, risk assessment, and policymaking simultaneously (production, evaluation and translation of science), particularly within European regulatory systems. This paper aims to address this gap by providing a cross-cutting analysis of these three domains and identifying actionable strategies to improve their alignment.

**FIGURE 1:**
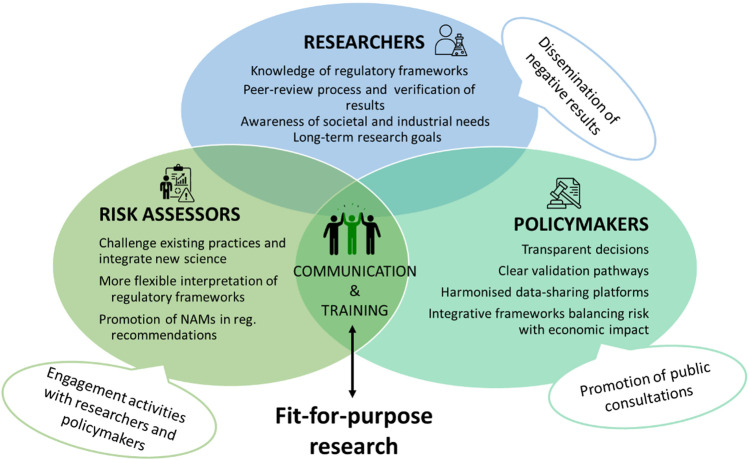
The role of researchers, policymakers, and risk assessors in bridging the gap between technological developments and regulation. This graphical representation illustrates how collaboration among these three key disciplines can facilitate the alignment of emerging technologies with regulatory frameworks, ensuring responsible and effective implementation.

This paper aims to identify the gaps currently observed in the three groups involved in translating research into regulation, and what can be done to foster better practices (Figure 1). It explores the hypothesis that misalignment between research and regulation results from differences in objectives, timelines, and limited mechanisms for integration. The analysis highlights how researchers, policymakers, and regulators can adopt specific practices to improve the uptake of science in regulatory processes. While other stakeholders, including industry, non-governmental organisations, and the public, are essential to the broader ecosystem, their roles fall outside the scope of the present analysis and warrant dedicated investigation.

## 2 Materials and methods

The review followed a semi-systematic approach, combining structured search strategies with expert-driven selection and interpretation. A semi-systematic targeted literature review was conducted in March 2025 to analyse the interfaces among researchers, regulatory agencies, and policymakers. The search was performed using databases including Web of Science, Scopus, and Google Scholar, complemented by grey literature sources such as reports from regulatory agencies (e.g., EFSA, ECHA, OECD) and policy documents from the European Commission. Emphasis was placed on examples from sectors where regulatory uncertainty has constrained innovation, such as novel foods, biotechnology, and human and environmental health. Insights from both academic and grey literature were synthesized to identify recurrent systemic challenges and evaluate proposed interventions from various national and supranational regulatory contexts.

Search terms included combinations of keywords such as “science-policy interface”, “regulatory science”, “risk assessment”, “evidence-based policymaking”, “data harmonization”, and “new approach methodologies (NAMs)”. Operators (AND, OR) were used to refine the search strategy.

Publications were screened to identify examples illustrating potential gaps between scientific research outputs and their regulatory or practical applicability. The evaluation of the selected literature was performed through expert judgement by the authors, based on their experience in regulatory science, risk assessment, and translational research. While this approach involves expert judgement, it is consistent with the objectives of a commentary article, which aims to provide interpretative and integrative insights rather than a fully systematic review. Inclusion criteria comprised publications addressing technical, regulatory, and social science perspectives relevant to the science–regulation interface, with a focus on European or comparable regulatory contexts. Exclusion criteria included studies lacking relevance to regulatory application or those focused exclusively on highly specialized technical outcomes without broader policy implications. The assessment focused on aspects such as clarity of regulatory relevance, methodological suitability for regulatory decision-making, and the extent to which studies addressed real-world implementation considerations. The literature was synthesised qualitatively through thematic analysis, identifying recurring patterns, systemic barriers, and proposed solutions across stakeholder groups.

The purpose of this assessment (particularly in Section 3) was not to evaluate individual authors or publications, but rather to identify recurring patterns and structural challenges in the interface between research and regulation. For this reason, specific publications used as illustrative examples in the internal assessment are not individually disclosed. Instead, the analysis focuses on aggregated observations and generalizable methodological issues observed across the screened literature.

Following this evidence synthesis, a set of actionable solutions was developed. These were assessed against three core criteria: feasibility within existing regulatory and institutional frameworks; cross-sector and cross-disciplinary relevance; and capacity to deliver long-term structural improvements in the science-regulation-policy interface.

The issues presented below are not ranked quantitatively, as the semi-systematic nature of the review did not allow for formal frequency analysis. However, they are broadly ordered based on their perceived relevance and recurrence across the reviewed literature and the authors’ expert experience. Additionally, their occurrence and impact are highly context-dependent and influenced by differing objectives. Instead, they are intended to illustrate recurring challenges observed across the literature, particularly when interpreted from a regulatory perspective.

## 3 Gaps between research and regulation: the role of the academia

### 3.1 Misleading titles and abstracts and misinterpretation of data

Misleading titles and abstracts often fail to accurately represent the study’s findings or intentions. Researchers, whether unintentionally or in an attempt to gain visibility, may use sensationalized, vague, or overstated language in titles and abstracts (Abdallah et al., 2024; Muhammed T and Mathew, 2022). This practice can distort the study’s true scope, results, or implications, leading to misconceptions among readers, including policymakers and regulators who may rely on these summaries for quick insights. For instance, title and abstract screening is an important step when carrying out a systematic review (Polanin et al., 2019) and the problem that misleading information at this step occurs extensively, can cause include for instance, false positives which will go to full-text screening, when maybe could have been excluded at the abstract screening step, implying a more time-consuming process, spending more human resources on the elaboration of systematic review, which might be the basis for subsequent research proposals or recommendations and policies (Wang et al., 2020; Dempster et al., 2022; Focosi et al., 2021).

While regulatory approaches often aim to protect vulnerable populations through conservative assumptions, challenges arise when findings from narrowly defined study populations are extrapolated without appropriate contextualisation. For example, results derived from specific subgroups may not capture variability across the general population or may lack sufficient detail to inform subgroup-specific risk assessments, highlighting the need for careful interpretation rather than direct generalization (Peters et al., 2024). This can result in regulatory policies that are not suitable for the broader population, potentially leading to ineffective or harmful outcomes. Similarly, specifying the health status of a human population in a clinical trial is essential, since the basal state might be completely different and results obtained following, for instance, oral consumption of a food or a drug, might not be reliable or relevant to assess safety or efficacy of those. On the other hand, ensuring that research findings are correctly contextualized and applied is essential for developing sound, evidence-based regulations, which is the responsibility of risk assessors and managers. A further example of misinterpretation of data, is in the context of statistics where after multiple statistical analyses only the desired outcome is presented without transparency about the number of tests run. One relevant role of risk assessors is, on top of identifying whether the statistical analysis is adequate, that a significant change is also representative of biological relevance (Committee, 2011; Levenson et al., 2023). The indistinct or improper use of statistical measures like the standard error of the mean and standard deviation, which are sometimes used interchangeably without clear indication, can potentially lead to misinterpretations of variability and precision.

Presenting findings without numerical data introduces subjectivity, hinders reproducibility, and complicates the evaluation of trends or statistical significance. Research should include both visual and numerical data for clarity and reliability. In this line, unethical practices, such as exaggerating results by altering scales (e.g., truncated y-axes) or selectively reporting data (e.g., ignoring the control group), can misrepresent outcomes (Driessen et al., 2022). Although there are tools such as WebPlotDigitizer (https://automeris.io/) that can help mitigate the repercussions of this issue, researchers are encouraged to transparently con fully share raw and fully quantifiable results.

### 3.2 Inadequate study designs and overlooked methodological and compliance standards

Publication of studies with significant design flaws, such as a lack of controls, small sample sizes, improper characterization of the test item employed not accounting for purity or presence of contaminants, the use of not reliable analytical methods, non-randomized sampling in human studies, or biases in data collection and analysis or improper statistical analysis, but presented as robust evidence, poses a serious issue in the scientific community (Fernandes-Taylor et al., 2011; Squeo et al., 2024).

Omission or poor discussion of the limitations of the methodology can lead to an overestimation of the reliability and applicability of the findings (Ott, 2024). For example, observational studies that suggest causal relationships without adequately accounting for confounding factors can mislead regulators into making decisions based on incomplete data (Ross and Bibler Zaidi, 2019). Misleading conclusions drawn from correlational data can result in inappropriate decisions for further research or recommendations (Gershman and Ullman, 2023).

Additionally, inconsistencies between the numbers reported in different sections raise concerns about reproducibility and data integrity. Incomplete reporting of experiments, and the use of multiple outcome measures for the same endpoint, without a clear rationale or explanation, contribute to the overall lack of transparency. Additionally, the lack of access to raw data or a detailed description of the methodology prevents independent verification of results. While authors may withhold certain details for future assessments or due to confidentiality concerns, it is essential that the data presented in the publication be sufficient to verify the conclusions. A similar issue arises with intellectual property, where the desire to protecting innovation may lead to withholding key data or methods. While this is understandable, it can limit regulatory applicability. Secure and confidential data-sharing channels can help balance innovation with the need for transparency.

Finally, in relation to how and where the report of scientific results is done, one challenge sometimes faced by regulators and scientists is that the same study might have different results in different publications (Altay and Koçak, 2021).

### 3.3 Selective reporting, publication bias and conflicts of interest

Selective reporting and publication bias present a significant problem in the research-regulation gap by distorting the overall evidence available to policymakers. When only positive results are published, while negative or inconclusive findings are ignored or suppressed, the resulting body of literature offers an incomplete view of the evidence (Stratton, 2016). As a result, regulators might base their decisions on biased results and policies may be implemented without considering the full spectrum of evidence. For instance, if policymakers use this incomplete evidence to evaluate a claim that consuming a certain food is linked to certain health benefits, their decisions might overestimate benefits or overlook potential risks, resulting in policies that do not reflect the true spectrum of scientific findings. It is responsibility of the risk assessors and managers to make sure that a complete picture of the literature is collected, and that is the reason why systematic reviews are usually preferred in risk assessments (O’Connor et al., 2017), although if negative results are not published, still will not be considered when systematically reviewing any specific topic.

In relation to conflict of interest, most journals have implemented the request for authors’ self-declaration of funding sources and perceived conflicts but sometimes conflicts of interest may not be clearly disclosed. This lack of transparency can lead to biased interpretations and misrepresented evidence. Addressing these conflicts of interest is crucial for ensuring that research informs regulation in a fair and unbiased manner (Dunn et al., 2016; Lundh et al., 2017).

### 3.4 Utilizing and reporting non-harmonized data in cross-national health comparisons

The use of non-harmonized data in cross-national health comparisons add complexity and if not handled properly can compromise the reliability and validity of health metrics. Discrepancies in data collection, surveys, and response scales across countries often result in inconsistencies that challenge the comparability of findings. For instance, variations in how long-term activity limitations or other health indicators are assessed.

However, even when harmonization aids comparability, it is a slow process that may hinder scientific progress. Integrating diverse datasets is complex due to variations in data collection and demographics. Researchers develop innovative methods to address these differences, but rigid harmonization can limit such advancements. Moreover, by the time standardized approaches are adopted, they may already be outdated, restricting methodological exploration. Strict harmonization also reduces data accessibility, potentially excluding valuable datasets and creating knowledge gaps. Developing harmonized datasets demands resources that may not be available to all researchers.

### 3.5 Best practices for carrying out responsible scientific research

In addressing the gap between research and regulation, it is essential for scientists to adopt comprehensive best practices that enhance clarity and responsibility when designing new projects and disseminating findings. The most obvious is to ensure the quality of the results through a proper quality control and quality assurance system. Requesting research laboratories, a level of control similar to regulatory good laboratory practices is unaffordable, but the basic principles can be adapted to the specific needs.

Early-stage studies, like preliminary clinical trials (small sample size, short-term interventions, insufficient endpoints assessed), provide valuable insights but often have limitations that must be transparently conveyed to prevent misinterpretation (Vossen, 2017). Designing an adequate experimental design from which, based on the results, different next steps could be taken in order to establish the future perspectives should be a priority for scientists (Michal et al., 2021).

It must be noted that the pressure to publish quickly has been further intensified by the rise of predatory journals and conferences held as business opportunities, which prioritize financial gain over the integrity of scientific work. This commercialization of academic publishing compromises the credibility of research, diminishing its relevance to the most current and significant scientific advancements (Richtig et al., 2018).

Journals and editors play a key role in enforcing responsible reporting by setting strict guidelines and rejecting submissions that use exaggerated language or omit study limitations. However, editorial biases, such as self-publication by editors-in-chief, can compromise these standards. Blind peer review can mitigate these biases by focusing on research quality rather than author reputation (Arumugam et al., 2020; Kozlov, 2024; Liu et al., 2023; Wykes and Parkinson, 2023).

Even when the priorities for academic researchers are triggered by policymakers, who decide on the funding programmes, there is an urgent need for the researchers to facilitate the verification of their results for allowing regulatory use, who should conduct comprehensive, peer-reviewed investigations that address these critical gaps and ultimately support the development of effective, science-based regulation (Fernandez et al., 2024; Landry et al., 2024; van Leeuwen et al., 2024).

## 4 Gaps between research and regulation: what can risk assessors do to help close this gap

### 4.1 Enhancing regulatory transparency and guidance

The lack of clear, structured guidance on how novel methodologies can be incorporated into risk assessment frameworks is key on this issue. This often results in uncertainty among researchers regarding regulatory expectations, leading to misaligned study designs and missed opportunities for impactful contributions. Risk assessors can play a crucial role by providing transparent guidelines that outline the criteria for integrating new scientific approaches into regulatory processes.

Beyond offering clear guidelines, regulatory bodies and their experts should actively promote the integration of research studies that are fit for purpose, rather than relying solely on previously validated studies from over 30 years ago (e.g. beta-cyfluthin Renewal Assessment Report (RAR), 2018; OECD Test No. 424: Neurotoxicity Study in Rodents, 1997 (EC, 2017; OECD, 1997) which may no longer align with current scientific and regulatory needs. The reliance on outdated methodologies slows progress and limits the adoption of innovative approaches that could provide more relevant, reliable, and humane alternatives. Risk assessors, have the competencies to identify which emerging scientific studies could be valuable for regulatory decision-making and should actively champion these studies in their recommendations. By doing so, they can initiate a natural path toward validation, ensuring that promising methodologies are considered early and assessed for their regulatory applicability.

### 4.2 Facilitating early regulatory involvement in research

The late-stage consideration of regulatory requirements in scientific studies often results in misaligned methodologies and wasted efforts. Risk assessors must take a more proactive role by engaging with researchers early in the research process. By participating in research projects from the conceptual stage they can help ensure that studies are designed with regulatory relevance in mind. This early alignment not only increases the chances of new methodologies and studies being accepted but also fosters a shared understanding between scientists and regulators.

Furthermore, regulatory agencies should promote the establishment of fellowship programs such as EU-FORA, that embed scientists within regulatory bodies, allowing them to gain first-hand experience with regulatory processes while also bringing fresh scientific perspectives into the regulatory space (Bronzwaer et al., 2016).

By facilitating early regulatory involvement in research, risk assessors can help shape studies in ways that make them more applicable for risk assessment, ultimately accelerating the integration of innovative approaches into regulatory frameworks.

### 4.3 Promotion and implementation of NAMs in regulatory recommendations

Regulatory agencies rely on proven, standardized methods to ensure consistency, reliability, and reproducibility of results. Such methods are essential for maintaining trust in regulatory decisions and ensuring public safety. Additionally, several regulatory frameworks, such as REACH (Registration, Evaluation, Authorisation and Restriction of Chemicals), require consideration of scientific publications as part of the assessment process. However, many NAMs remain at experimental stages, pending the rigorous validation implemented under regulatory frameworks. Strict interpretation of formal validation creates a disconnect between science and regulatory practice. Consequently, opportunities for the timely integration of these innovative approaches into regulatory frameworks are delayed or missed entirely (Cattaneo et al., 2023). This highlights a fundamental tension between maintaining trust in regulatory decisions, which relies on standardized and validated approaches, and enabling flexibility to incorporate emerging scientific methods. Addressing this tension requires transparent criteria for evaluating new evidence and clear communication about uncertainty.

Currently, mechanisms exist within regulatory frameworks to review and incorporate NAMs, yet they remain underutilized for fostering innovation. For example, the Testing Proposals under REACH regulation and under the Transparency Regulation (European Commission, 2019) require a preassessment of the study design by experts in the regulatory agencies’ committees, that has not yet been employed to promote new approaches or alternative methodologies. This mechanism could be reimagined to prioritize innovation by tasking committees to consider alternatives to traditional studies that could better fulfil the information gap (Čavoški et al., 2025).

The current validation process for novel approaches follows a rigid framework, requiring significant time and efforts. A shift in perspective is needed. There are benefits in validation, particularly in ensuring relevance and incorporating principles for assessing reliability (e.g., a validated OECD Guideline study under Good Laboratory Practices (GLP) is not necessarily reliable, but its reliability can be verified). However, particularly for NAMs, a validation process requiring over 10 years means that the methodology is often obsolete by the time it is validated.

Historically, regulatory science began with the use of non-validated studies, which were frequently employed due to their relevance and proven utility within the regulatory framework. Validation and standardization followed after these studies demonstrated consistent application and reliability, providing clear indications to the regulated sector on the studies to be conducted and facilitating mutual recognition among jurisdictions. Regulatory bodies could adopt a more pragmatic approach for NAMs. Instead of waiting for full validation prior to their use, regulators could focus on the immediate relevance, scientific validity, and applicability of these methods to address gaps in the regulatory framework, rather than relying solely on standardized methods. The European Medicines Agency (EMA) and Food and Drug Administration (FDA) fit-for-purpose qualification should also be mentioned as an essential mechanism for evaluating the scientific merit of emerging methodologies, offering proposals for their scientific validity. Studies designed according to identified regulatory needs could then be retrospectively validated based on their successful application in regulatory decision-making.

This would promote a culture of adaptive regulation, accelerate the adoption of alternative methods and fostering a regulatory landscape that supports both scientific developments and public safety.

### 4.4 Encouraging the use of weight-of-evidence approaches to integrate diverse data sources

The integration of NGRA and updated studies into regulatory frameworks necessitates a comprehensive evaluation of diverse data sources. EFSA has developed guidance on the use of the weight-of-evidence (WoE) approach in scientific assessments, providing a structured framework for this purpose (Hardy et al., 2017). The WoE approach involves a systematic process comprising three key steps: assembling evidence by collecting relevant data and organizing it into lines of evidence based on similarity, weighing evidence assessing each line of evidence for reliability, relevance, and consistency, and integrating evidence combining the evaluated lines of evidence to form a comprehensive assessment (Martin et al., 2018).

By adopting the WoE approach, regulatory bodies can systematically evaluate and integrate data from various sources, thereby enhancing the robustness and transparency of decision-making processes. This method allows for a balanced consideration of all available information, facilitating the acceptance and implementation of innovative methodologies within regulatory frameworks. The ECHA also employs the WoE principle, as outlined in their Practical Guide - How to use alternatives to animal testing to fulfil your information requirements for REACH registration (ECHA, 2016).

### 4.5 Encouraging the use of real-world evidence (RWE)

Traditional regulatory decisions often rely on controlled laboratory studies, which, while valuable, may not fully capture the complexities of human exposure, variability, and real-world conditions (Burns et al., 2022; Prilla et al., 2024). Risk assessors can advocate for the incorporation of real-world variability in their risk assessment. Data from human biomonitoring, epidemiological studies, and realistic exposure assessments addressing variability should be used to benchmark the pre-marketing authorization assessments and associated regulatory decision-making and incorporated in post-market assessments. These data sources provide a more accurate reflection of actual risks, improving the relevance and applicability of regulatory evaluations.

To facilitate this integration, regulatory agencies should promote the flexible implementation of current frameworks, particularly in terms of evidence integration, methodological pathways, and iterative assessment processes, while maintaining fixed regulatory protection goals. Rather than rigidly adhering to predefined designs, risk assessors can encourage adaptive methodologies, within tiered risk assessment approaches, which allow evolving scientific methodologies to be incorporated as they emerge (Committee, 2021). By enabling a structured yet dynamic evaluation process, regulators can ensure that innovative research contributes meaningfully to safety assessments without unnecessary delays due to outdated validation procedures. Operationally, this could include pilot schemes for integrating NAMs in parallel with traditional methods, formal guidance on the use of probabilistic approaches, and structured pathways for early dialogue between researchers and regulators during study design.

Moreover, leveraging real-world data and big data analytics can enhance the robustness of regulatory decisions. Advances in data science, including machine learning and artificial intelligence, offer powerful tools for analysing large-scale datasets, identifying patterns, and refining risk assessments.

### 4.6 Stakeholder engagement and training

The use of platforms where all relevant stakeholders are incorporated (industry experts, patient advocates, non-profit organisations, etc.), fosters engagement between the different entities and risk assessors have the power to facilitate these exchanges. A revision on stakeholder participation could create a more inclusive and responsive framework that integrates emerging research while better addressing ethical, social, and public health considerations.

To maintain trust in regulatory processes, committees and panels must promote transparency and mitigate conflicts of interest in their recommendations and decisions. Clear guidelines should be established to ensure that scientific evaluations are driven by robust, unbiased evidence rather than external pressures. Risk assessors can advocate for greater transparency by encouraging open discussions, public reporting of detailed rationales, and independent reviews of regulatory recommendations.

Additionally, researchers often develop innovative methodologies without a clear understanding of how to align their work with regulatory requirements, resulting in valuable scientific advancements being overlooked. Regulatory agencies can address this by providing targeted training programs for researchers, equipping them with the knowledge needed to design studies that meet regulatory expectations. These programs should also extend to experts on panels and committees, ensuring that risk assessors stay informed about cutting-edge scientific developments.

### 4.7 Addressing funding and incentives for regulatory-oriented research

Much of the scientific research produced today is driven by academic interests, economic priorities, or broader innovation goals, which may not always align with the practical requirements of regulatory agencies. Risk assessors can help bridge this gap by advocating for funding programs that prioritize regulatory-relevant research, ensuring that financial resources are directed toward studies that can inform and improve risk assessment processes (e.g. PARC (https://www.eu-parc.eu/) or ASPIS (https://aspis-cluster.eu/)).

Regulatory agencies have a key role in shaping funding priorities by identifying pressing regulatory gaps and promoting research agendas that address them (e.g. Key Areas of Regulatory Challenge by ECHA (ECHA, 2023)). It is crucial that such agencies actively engage on the project development to ensure that such alignment with their expectations is obtained and that the project will be practically implementable for regulatory purposes (e.g., development and validation of methodologies aligned with current regulatory challenges). Moreover, they can establish financial incentives, such as grants, fellowships, or public-private partnerships, to encourage researchers to conduct studies that fill critical knowledge gaps. Several agencies have already implemented such approaches. For example, national bodies such as ANSES (France) and European initiatives under Horizon Europe allocate dedicated funding to address regulatory research gaps, illustrating that this transition is already underway and can be further expanded.

An important aspect not often discussed is the development of test guidelines at the international level, particularly within the OECD framework. The process of achieving consensus among member countries, while essential for harmonisation and mutual acceptance of data, can significantly delay the incorporation of new scientific knowledge into regulatory practice. Additionally, transparency regarding potential conflicts of interest among experts involved in guideline development varies across contexts, which may influence both the pace and direction of methodological updates. Addressing these challenges is critical for ensuring that regulatory science remains both robust and responsive to innovation.

## 5 Gaps between research and regulation: what can policymakers do to help close this gap

### 5.1 Homogenised, trusted and transparent data sharing platforms

The establishment of homogenized, trusted, and transparent data-sharing platforms can ensure that data from diverse sources is interoperable, credible, and readily accessible for both scientific research and risk assessors (Nature Portfolio, 2024; Pearson, 2024).

Transparent data-sharing platforms serve as a vital interface between research and regulation by enabling (1) standardization, where policymakers can mandate common reporting standards ensuring that data generated by researchers aligns with regulatory requirements, (2) interoperability by fostering harmonized frameworks, making it easier to integrate data from diverse sources, enhancing its utility for cross-disciplinary and cross-regulatory applications, and (3) validation with centralized platforms that provide mechanisms for validating new methodologies, thereby improving their credibility and acceptance in regulatory contexts. To close the gap between research and regulation, policymakers can take the following measures:

- Promote collaborative platforms: facilitating the co-creation of standards and practices that align research outputs with policy needs.
- Establish open and innovative approaches (see Section 4.2).
- Support training and education (see Section 5).
- Incentivize transparency by providing funding for projects that prioritize open data and transparency (see Section 4.4) (Smits et al., 2025).

Therefore, open-access databases that are in accordance with the findable, accessible, interoperable, and reusable (FAIR) guiding principles, are of interest for the scientific community and regulators. Promoting their use by researchers to include their generated data and facilitating the update of the state-of-the-art of specific research topics allows for a more complete data analysis, instead of relying on constant literature reviews (Ke et al., 2024).

It must be acknowledged how European Regulation took a significant step forward by implementing the Transparency Regulation (European Commission, 2019). In this regard, for regulated products in the food chain, applicants have to notify certain studies before their start date and to submit in the application all relevant notified studies (for commissioned studies conducted to support an application). This reduces selective reporting, ensures comprehensive evaluations, and strengthens scientific rigor.

Another crucial aspect is the public consultations in risk assessment. While public consultations offer a transparent platform for sharing scientific input on specific regulatory areas, they often fall short in capturing contributions from the broader research community. Despite being open invitations for scientific engagement, these consultations frequently receive minimal input from researchers, particularly in the form of empirical data or unpublished findings (e.g. virgin olive oil health claim (EFSA-Q-2024-00470), formaldehyde (CLH-O-0000007130-88-01/F)). This gap is not necessarily due to a lack of willingness to contribute, but rather a widespread unawareness that such consultations are taking place. Many researchers are simply not informed of these opportunities or do not perceive them as relevant to their work. This lack of visibility undermines the potential for evidence-based policy development and reinforces the disconnection between scientific advancements and regulatory processes. It is essential to enhance the visibility and accessibility of public consultations within the research ecosystem. Doing so would increase awareness and participation, enabling the regulatory process to benefit more fully from current scientific knowledge and data.

### 5.2 Creating open and innovative approaches

Fragmented global regulatory landscapes hinder innovation and sustainable practices by creating inconsistent standards for safety and environmental performance. Companies often struggle to navigate these diverging frameworks, which lead to redundancy and inefficiency.

Standardization initiatives by internationally recognised bodies provide clarity for companies, allowing them to focus on developing sustainable innovations without the burden of managing fragmented regulatory systems. Aligning regulatory expectations across regions encourages global collaboration and accelerates the adoption of best practices. An example of that is the INFOGEST protocol, an *in vitro* methodology for evaluation of protein digestibility, which has been internationally harmonized and recognized, but still pending its validation, currently under assessment by ISO (Krul et al., 2024; Santos-Sánchez et al., 2024). However, this validation still might not imply regulatory acceptance, depending on the biological relevance of the outcomes (Sections 3.1, 3.2).

However, harmonizing regulatory protocols does not necessarily resolve all challenges. Many existing frameworks are outdated and no longer reflect contemporary scientific advancements. For example, current legislation often originates from paradigms established in the 1950s, preventing the adoption of newer methods and technologies. A good initiative to counteract this is the regulatory sandboxes (https://www.eit.europa.eu/our-activities/opportunities/digital-sandbox-accelerator), which are controlled policy environments that enable the testing of innovative technologies or practices under the supervision of regulatory bodies in which there is mutual learning between both parties. However, regulatory sandboxes face limitations, including issues of scalability, potential regulatory fragmentation, unequal access for smaller actors, and challenges in ensuring long-term compliance once innovations exit the sandbox context.

An additional complication arises from the fact that while regulatory guidelines are updated regularly, the reassessments are mostly based on reference legacy *in vivo* studies that cannot be reconducted with the modernized standards due to ethical concerns. This is particularly evident in sectors with periodic re-assessments such as pesticides, where vertebrate studies conducted in the 1970s remain a cornerstone for regulatory evaluation. This may be also relevant for *in silico* methods, such as read-across and quantitative structure-activity relationships (QSAR), that rely on extrapolated data from these outdated *in vivo* studies. This reliance represents a significant limitation, as it constrains the potential of regulatory science to fully embrace modern alternatives and more ethical approaches.

Ultimately, addressing these issues requires a multi-faceted strategy that combines the harmonization of regulatory systems, the modernization of legal frameworks, and the validation of NAMs to replace outdated methods. Such efforts are crucial for fostering innovation while ensuring that safety, ethical, and environmental standards are upheld across global industries. The opportunity is the integration of all existing data, facilitating the regulatory use of different information sources in a consistent manner, such as the Integrated Approaches to Testing and Assessment (IATA) developed by the OECD. Transferring IATA case studies into assessments under the regulatory framework should be considered among the regulatory priorities for chemical safety assessments.

### 5.3 Stakeholder collaboration

Stakeholder collaboration between industry, policymakers, risk assessors, and researchers is essential to align innovation with societal needs and regulatory frameworks (D’Este et al., 2019). Industry, as the end-user of research developments, brings valuable insights into practical applications and market feasibility, while policymakers articulate priorities and regulatory expectations to ensure public safety and societal benefit. Risk assessors ensure that regulatory decisions are grounded in scientific rigor and transparency, bringing expertise in risk evaluation, and balancing innovation with safety (Abt et al., 2010). Researchers, in turn, define the state of scientific knowledge and the feasibility of proposed innovations. Collaborative platforms, such as joint task forces, cross-sector workshops, and innovation consortia, can help these stakeholders identify shared goals, anticipate challenges, and co-create solutions.

An important issue to discuss is the reluctance of some industry players to share data and research due to intellectual property (IP) concerns. While these concerns are valid, limited data sharing can hinder risk assessment, as assessors are unable to access emerging technologies or evaluate potential risks comprehensively. This can create blind spots in regulatory science and delays the integration of innovative solutions (companies may withhold detailed mode-of-action studies or environmental impact data), potentially limiting comprehensive risk evaluation for regulators or for the broader scientific community to evaluate long-term implications (de Sandes-Guimarães et al., 2022).

### 5.4 Funding and incentives for transparent and harmonized data practices

Many researchers rely on project-based funding that pressures them to produce specific, often positive results, rather than focusing on rigorous, unbiased data collection. Additionally, there is little motivation for researchers to contribute their data to open databases, as they gain no direct academic or professional benefits from doing so. Policymakers can address these challenges by providing funding and incentives to practices that promote transparency and harmonization.

In this regard, Horizon Europe is the EU’s flagship research and innovation programme, designed to drive scientific excellence, support industrial competitiveness, and tackle global challenges through targeted funding. Building on its predecessors (such as Horizon 2020), Horizon Europe funds projects across a wide range of thematic areas with the overarching goal of fostering innovation that aligns with EU policy priorities, including the Green Deal, Farm to Fork Strategy, and digital transition. Beneficiaries are required to provide open access to scientific publications and, where possible, to underlying data (Slepak and Pozhilova, 2021).

The horizon scanning exercise is a proactive method used to identify and evaluate emerging trends, risks, and opportunities that could significantly impact future policies, research, or industries. This process is vital for anticipating changes in technology, science, and society, and it allows decision-makers to prepare for potential challenges or capitalize on upcoming developments. In the context of EU research and policy, horizon scanning plays a crucial role in informing the design of programs like Horizon Europe (Ballester et al., 2023; Paulović et al., 2022).

However, horizon scanning exercises face challenges such as uncertainty in predicting future developments, limited scope of data, resource constraints, difficulty translating findings into actionable policies, and insufficient stakeholder engagement, which can lead to incomplete analyses, biases, and slow adaptation to new information, ultimately limiting their effectiveness in informing strategic decision-making (Baldwin et al., 2022).

## 6 The key element: the importance of open communication and targeted training

Open communication between researchers, regulators, and policymakers is essential to align scientific outputs with regulatory needs. Structured interactions such as workshops, advisory panels, and stakeholder consultations can facilitate mutual understanding and improve the relevance of research for regulatory decision-making. Complementary to this, targeted training initiatives for both researchers and regulators are critical to bridge knowledge gaps, enhance methodological rigor, and support the effective translation of science into policy (EC et al., 2022).

These panels can assist in identifying relevant studies and contextualizing findings, thus enhancing the regulatory review process. Moreover, such panels, including external experts, could be leveraged to explore alternatives to traditional *in vivo* studies by requiring a comprehensive battery of NAMs and new studies that could be nowadays, more fit to the current societal demands, such as better connection of risk estimation with public health under real-world conditions. In this line, for example a recent EFSA editorial (EFSA, 2025) emphasizes the need to strengthen regulatory science through targeted research and innovation, particularly in areas like NAMs, environmental risk assessment, and allergenicity. Similarly, EFSA is promoting stakeholder meetings on topics of scientific interest (Afonso et al., 2024) and launching a stakeholder group to improve the risk assessment process. In this regard, authorities often publish calls for tenders in relation to their activities, such as the “Ensuring EFSA’s readiness to address risk assessment needs related to food and feed innovation” (EFSA/2025/OP/0007-PIN) or “Scientific and technical support work related to hazard assessment and identification, including regulatory support of work on CLP, Dossier and Substance Evaluation, POPs, DWD and future tasks under certain water protection directives” (ECHA/2024/OP/0004), for example.

Developing evaluation criteria covering study design, statistical rigor, and conflict of interest disclosures, ensures consistency and robustness in regulatory assessments. Prioritizing systematic reviews (SRs) and meta-analyses further strengthens decision-making by providing comprehensive insights beyond isolated studies (Guzelian et al., 2005). Nonetheless, EFSA has adopted SRs for decision-making but emphasizes their thoughtful use due to time constraints. SRs are particularly useful for controversial topics or areas with expert disagreements, ensuring transparency. While full SRs may not always be feasible under tight deadlines, rapid reviews can be considered but are less comprehensive (O’Connor et al., 2017).

Encouraging researchers to publish in Open Access (OA) has been a priority for the European Commission, and in recent years this practice has been implemented (RTD, 2016), although there is still a significant number of publications not following this scheme sometimes driven by the high costs of publishing OA.

By adopting these recommendations, regulatory agencies can significantly improve their processes for scrutinizing literature. Enhancing critical appraisal skills, developing robust guidelines, and promoting collaboration with the research community are crucial steps in ensuring that regulatory decisions are informed by high-quality evidence. In doing so, regulators can better protect public health and safety while supporting scientific developments. However, it must be noted that regulatory agencies often face other problems that complicate their efforts to bring research to regulation. For instance, the challenge of data harmonization makes it difficult to integrate findings from diverse studies, leading to inconsistencies and gaps in understanding. Additionally, regulators frequently encounter the rapid advancement of technologies that evolve faster than regulatory frameworks can be updated, creating gaps where no single entity is responsible for addressing the disconnect between innovation and legal standards (e.g., the use of artificial intelligence). To ensure that research and development activities can be useful for regulatory frameworks, it is essential to identify the specific research gaps that currently have limited or no peer-reviewed, published studies (e.g., long-term effects of novel synthetic chemicals under real-world exposure scenarios, or the evaluating the socio-economic and health impacts of new technologies). These gaps present significant challenges for regulators, who rely on robust, evidence-based data to establish guidelines and make policy decisions. The lack of high-quality, targeted research in these areas not only impedes the timely development of regulatory standards but also risks public and environmental safety, and for that, communication among the different parties is essential. Furthermore, the lack of cross-disciplinary integration can hinder the development of cohesive policies, as regulations often require insights from various fields, such as toxicology, engineering, and economics, yet research is frequently isolated.

To effectively align scientific developments with regulatory processes and societal expectations, it is crucial to understand the distinct roles and perspectives of key stakeholder groups: researchers, risk assessors, and policymakers. Each group contributes uniquely to the science-policy interface, yet they also face specific needs and challenges that can impact collaboration and progress (Table 1). The table below summarizes the main activities carried out by each group, their core needs for fulfilling their roles, the challenges they commonly encounter, and recommendations for improving cooperation, efficiency, and impact. This structured overview is intended to support a more integrated approach to research-driven regulation and evidence-based policymaking.

**TABLE 1:** Overview of stakeholder roles, needs, challenges, and opportunities for improvement.

Category	Researchers	Risk assessors	Policymakers
Main activities	Conduct experiments and generate data Develop methods and tools Publish findings	Analyze and interpret scientific data Assess risks based on evidence Prepare technical reports	Develop and implement policies Make decisions based on risk assessments Engage stakeholders
Main needs	Funding for research and open access publication Access to updated tools and methodologies	Access to high-quality and reliable data Collaboration with researchers and with risk managers Clear indication of regulatory needs Opportunities for following scientific developments	Clear and actionable assessments Public acceptance and communication strategies Integration of political and regulatory frameworks
Main challenges faced	Limited funding and resources Gaps in communication with assessors and managers Pressure to publish over practical impact	Incomplete or uncertain data Regulatory pressure Political or industrial pressure on impartiality Pressure for timely delivery of assessments	Conflicting stakeholder interests Uncertainty in long-term outcomes of policies Public mistrust in decisions
Points for improvement	Knowledge of regulatory frameworks Peer-review process and verification of results Awareness of societal and industrial needs Long-term research goals Dissemination of negative results	More flexible interpretation of regulatory frameworks Openness for challenging existing practices and integrating new science Training on new scientific tools and methodologies Engagement activities with researchers and policymakers	Integrative frameworks balancing risk with economic impact at relevant levels Effective crisis management plans Transparency and accountability Promotion of public consultations

## 7 Conclusion

Bridging the gap between scientific research and regulatory frameworks is crucial to ensure that advancements in science effectively inform policy and public health decisions. This paper has identified key challenges that contribute to this disconnect, including methodological inconsistencies, misaligned priorities, and insufficient communication between researchers, risk assessors, and policymakers. These challenges undermine the reliability, relevance, and practical applicability of research findings, ultimately weakening regulatory decision-making and diminishing public trust.

To address these issues, fostering interdisciplinary collaboration and promoting targeted training are essential. Researchers play a pivotal role in generating high-quality, reproducible data and methodologies, yet they often face constraints such as limited funding, pressure to publish, and insufficient awareness of regulatory needs. Strengthening researchers’ understanding of regulatory frameworks and societal expectations can enhance the applicability of scientific findings. Additionally, adopting transparent reporting practices, minimizing selective reporting and publication bias, and ensuring realistic interpretation of results will improve the utility of research for regulatory purposes.

Risk assessors serve as the bridge between scientific research and regulatory decision-making, ensuring that risk evaluations are based on reliable, standardized methodologies, while being open to adopt new methodologies pending validation but showing scientific credibility and relevance. To enhance the applicability of scientific findings, risk assessors must advocate for the adoption of consistent assessment frameworks, promoting guidelines that align with both academic rigor and regulatory feasibility, without forgetting that new methodologies are appearing and could be also relevant for risk assessment. Additionally, fostering interdisciplinary collaboration is essential to refine hazard characterization and risk quantification, reducing ambiguities that hinder policy implementation. By integrating transparent, reproducible, and systematic evaluation processes, and the use of all available scientific knowledge and tools, risk assessors contribute to strengthening the trustworthiness of regulatory decisions while facilitating the translation of scientific advancements into actionable policies.

Policymakers are central to narrowing the divide between scientific innovation and regulatory frameworks. Ensuring transparency, harmonized data-sharing platforms, and evidence-based decision-making can enhance regulatory efficiency and public trust. Addressing conflicting stakeholder interests, uncertainty in long-term policy outcomes, and public scepticism requires clear validation pathways, effective risk communication strategies, and funding initiatives that support high-quality, reproducible research. Additionally, aligning policy development with scientific advancements through structured engagement with researchers and risk assessors will improve the integration of science into regulation.

A key element in bridging these gaps is the promotion of open communication and targeted training across all stakeholder groups. Researchers, risk assessors, and policymakers must engage in continuous dialogue to ensure that scientific findings are interpreted and applied effectively. Training initiatives should focus on regulatory requirements, methodological transparency, and crisis management strategies, fostering a shared understanding of priorities and expectations.

By integrating transparent, reproducible, and systematic evaluation processes, but accounting for the continuous evolution of knowledge and methods, scientific and regulatory communities can strengthen public confidence in science while promoting innovation and effective policymaking. This collaborative approach will enhance the integrity of research, improve its societal and regulatory impact, and set a precedent for future scientific and policy developments. Ultimately, the insights presented in this paper have the potential to influence researchers, regulators, publishers, and the public, fostering a more responsible and transparent scientific environment.

This work has several limitations. The semi-systematic and interpretative nature of the analysis may introduce subjectivity, and the absence of formal quantitative synthesis limits the ability to rank identified challenges. Additionally, while efforts were made to include diverse sources, some relevant literature may not have been captured. The focus on European regulatory contexts may also limit generalisability to other jurisdictions. Despite these limitations, the study provides a structured overview of key challenges and opportunities at the science–regulation–policy interface.
